# Inflammatory Reprogramming Mediates Changes in Three-Dimensional Strain Capacity and Cardiac Function in Beagle Dogs with Doxorubicin-Related Cardiomyopathy

**DOI:** 10.31083/j.rcm2502062

**Published:** 2024-02-18

**Authors:** Yifan Chen, Yihui Shen, Hui Zhang, Xuejun Wang, Yuchen Xu, Jian Zhang, Weiguang Zhao, Rui Zhao, Zhihong Liu, Leilei Cheng, Junbo Ge

**Affiliations:** ^1^Department of Cardiology, Zhongshan Hospital, Fudan University, Shanghai Institute of Cardiovascular Diseases, Key Laboratory of Viral Heart Diseases, National Health Commission, 200032 Shanghai, China; ^2^Department of Echocardiography, Zhongshan Hospital, Fudan University, Shanghai Institute of Cardiovascular Diseases, Shanghai Institute of Medical Imaging, 200032 Shanghai, China; ^3^Department of Urology, Shanghai General Hospital, Shanghai Jiao Tong University School of Medicine, 200080 Shanghai, China

**Keywords:** doxorubicin (DOX), inflammatory reprogramming, cardiotoxicity, cardio-oncology, RT3DE, beagle dog

## Abstract

**Background:**

The cardiotoxicity of doxorubicin (DOX) limits its use in cancer treatment. To address this limitation, we developed a novel animal model that uses beagle dogs to investigate DOX-induced cardiac disorders. Unfortunately, the lack of effective cardioprotection strategies against DOX-induced cardiotoxicity poses a significant challenge. To establish a canine model for low-mortality DOX-induced cardiac dysfunction and explore the relationship between inflammatory reprogramming and DOX-related cardiotoxicity.

**Methods:**

Twenty male beagle dogs aged two years were randomly assigned into the DOX (N = 10) and control (CON) (N = 10) groups. DOX was infused (1.5 mg/kg) every two weeks until doses cumulatively reached 12 mg/kg. Serum biomarkers and myocardial pathology were evaluated, while real-time fluorescence-based quantitative polymerase chain reaction (RTFQ-PCR), two- and three-dimensional echocardiography (2DE and RT3DE), functional enrichment, and matrix correlation were also performed.

**Results:**

In the DOX group, high-sensitive cardiac troponin T (hs cTnT) and N-terminal pro-brain natriuretic peptide (NT-proBNP) were significantly increased. Myocardial pathology indicated early to medium myocardial degeneration via a decreased cardiomyocyte cross-sectional area (CSA). Increased levels of inflammatory gene transcripts (interleukin 6 (IL6), tumor necrosis factor (TNF), transforming growth factor β (TGFβ), intercellular adhesion molecule 1 (ICAM1), interleukin 1 (IL1), interleukin 1β (IL1β), and interleukin 8 (IL8)), of collagen metabolism and deposition regulatory genes (matrix metalloproteinase (MMP) family and tissue inhibitor of matrix metalloproteinase (TIMP) family), and the natriuretic peptide family (NPS) (natriuretic peptide A, B and C (NPPA, NPPB, and NPPC)) were observed. Strain abnormalities in the right ventricular longitudinal septal strain (RVLSS), right ventricular longitudinal free-wall strain (RVLFS), left ventricular global longitudinal strain (LVGLS), and left ventricular global circumferential strain (LVGCS) were detected at week 28 (vs. week 0 or CON group, *p* < 0.05, respectively). A significant decline in RVLSS and RVLFS occurred at week 16, which was earlier than in the corresponding left ventricular areas. A significant right ventricular ejection fraction (RVEF) decline was noted at week 16 (vs. week 0, 33.92 ± 3.59% vs. 38.58 ± 3.58%, *p* < 0.05), which was 12 weeks earlier than for the left ventricular ejection fraction (LVEF), which occurred at week 28 (vs. week 0, 49.02 ± 2.07% vs. 54.26 ± 4.38%, *p* < 0.01). The right ventricular strain and functional damages correlated stronger with inflammatory reprogramming (most R from 0.60 to 0.90) than the left ones (most R from 0.30 to 0.65), thereby indicating a more pronounced correlation.

**Conclusions:**

Inflammatory reprogramming mediated disorders of strain capacity and cardiac function predominantly in the right side of the heart in the newly established DOX-related cardiomyopathy beagle dog model.

## 1. Introduction

Doxorubicin (DOX), a prototype agent of anthracycline, has been proven to be efficacious against a wide range of malignant neoplasms [1]. However, its dose-dependent chronic and irreversible cardiotoxicity [2] has limited its clinical application, thus, close surveillance should be applied to its use [3].

It is pivotal to establish an animal model that can be used to explore mechanisms and clinical strategies. Previous anthracycline-related toxicity models on small animals [4, 5, 6] were all inferior in reflecting echocardiographic and hemodynamic alterations since their hearts were too small to be accurately assessed. Beagle dogs can be monitored using the aforementioned indicators, and therefore, represent an improvement on previous methods [5, 7]. Thus, we established a simple and safe new model for DOX-induced cardiac disorder in beagle dogs.

Unfortunately, effective protections for DOX-induced cardiotoxicity are still lacking. Bisdioxopiperazine dexrazoxane (ICRF-187), an iron-once chelator, is the only approved cardio-protectant that targets anthracyclines-related congestive heart failure [8]; however, its use has been limited owing to its induction of secondary malignancies (SMNs) [9].

Inflammatory reprogramming is reportedly associated with myocardial remodeling, which in turn affects the cardiac function of a variety of cardiomyopathies [10, 11]. This previous finding prompted our study to explore the relationship between inflammatory reprogramming and DOX-induced cardiomyopathy in this new beagle dog model, to identify further mechanisms and treatments.

## 2. Methods 

### 2.1 Animal Protocols

Twenty male beagle dogs aged two years, weighing 23–28 kg, were obtained from Jambo Biological Technology Co., Ltd (Shanghai, China). They were randomized using an online system (https://www.lifeguideonline.org) and placed into the DOX or control (CON) groups in a 1:1 ratio. Prior to commencing the experiment, pre-existing cardiac conditions were excluded in both groups, including pulmonary stenosis. All animals were individually housed in clean cages and were provided with free water and food. Indwelling needles (24G) were used to establish intravenous access through the medial cephalic vein of the forelimb for Telazol anesthesia (10 mg/kg, intravenously) and through the saphenous vein in the hind limb for DOX or 0.9% sodium chloride injection.

**Fig. 1.**
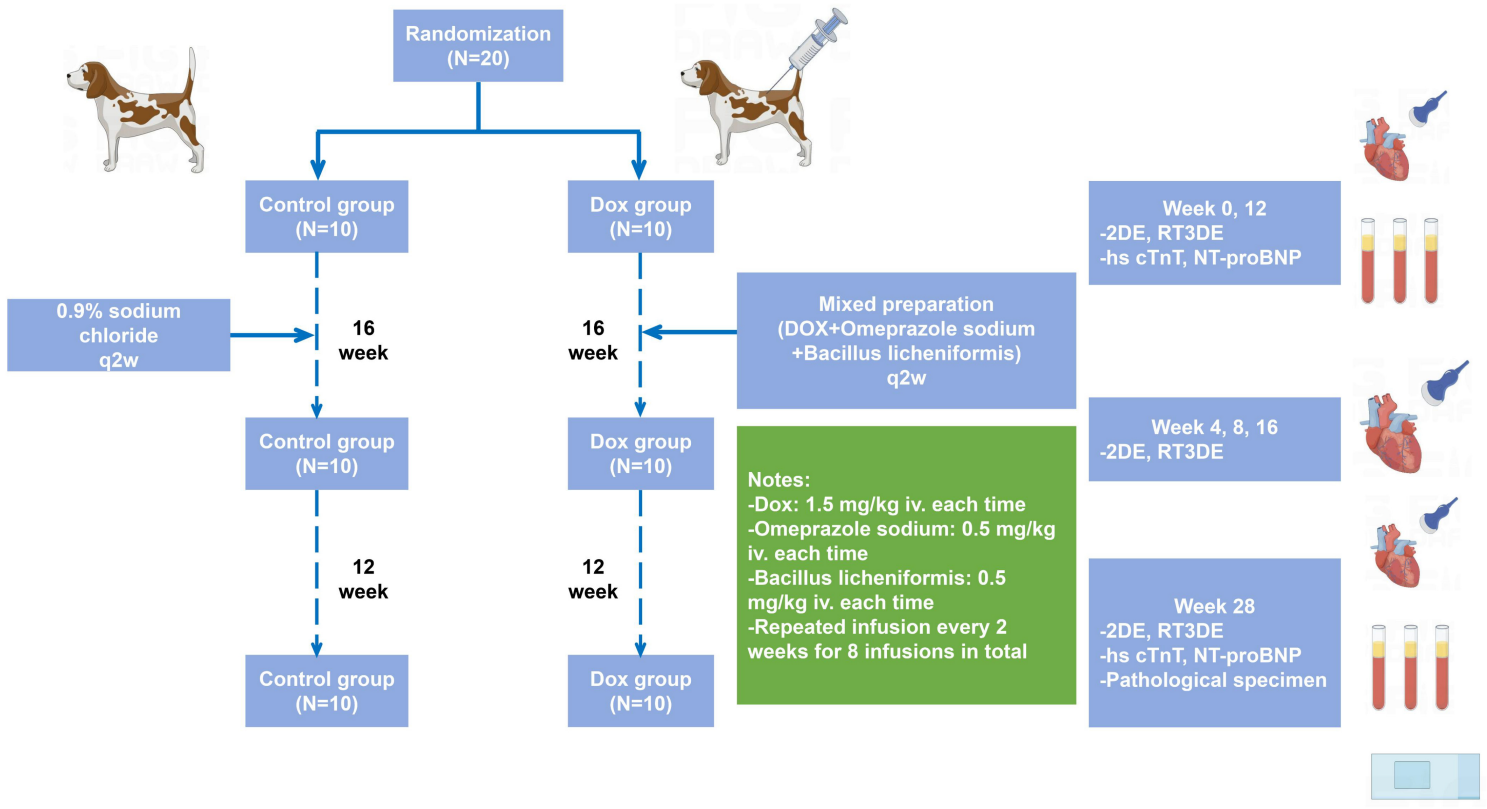
**Study protocol of the new DOX-related cardiomyopathy beagle dog model**. DOX, doxorubicin; iv., intravenous administration; q2w, every 2 weeks; 2DE, two-dimensional echocardiography; RT3DE, real-time three-dimensional echocardiography; hs cTnT, high-sensitive serum cardiac troponin T; NT-proBNP, N-terminal pro-brain natriuretic peptide; N = 10 dogs per group.

Owing to the fatal DOX-induced gastrointestinal bleeding (unpublished data: **Supplementary Fig . 1**), adequate attention was required to add gastrointestinal protective agents during DOX administration. In this protocol, a digestive tract protective mixed pharmaceutic preparation was applied for DOX hydrochloride (EnergyChemical, Shanghai, China), omeprazole sodium for injection (Luoxin Pharmaceutical, Shandong, China), and Bacillus licheniformis (Jingxin Pharmaceutical, Zhejiang, China). Subjects allocated to the DOX group received a 30 min intravenous infusion mixture of 1.5 mg/kg DOX and both 0.5 mg/kg omeprazole sodium and Bacillus licheniformis dissolved in 250 mL 0.9% sodium chloride. Synchronously, the control group received an injection of only 250 mL of 0.9% sodium chloride. Repeated infusion was performed every two weeks for a total of eight infusions (Fig. 1).

All animals participated in the study with signed owner consent and the protocols were approved by the Animal Ethics Committee of Zhongshan Hospital, Fudan University.

### 2.2 Biochemical Determination

Whole blood samples (15 to 25 mL) were drawn from the saphenous vein of the hind limb before anesthesia at weeks 0, 12, and 28 in both groups, left to stand at room temperature for 2 hours, and then centrifuged at 1000 g for 20 minutes to collect the serum. The serum levels of high-sensitive cardiac troponin T (hs cTnT) and N-terminal pro-brain natriuretic peptide (NT-proBNP) were determined to assess cardiomyocyte damage and myocardial stretch, respectively, using available enzyme-linked immunosorbent assay (ELISA) kits (Ze Ye Biological Technology Co., Ltd., Shanghai, China). Optical density (OD) values were measured for each sample at a wavelength of 490 nm (for hs cTnT) or 450 nm (for NT-proBNP) using an enzyme immunoassay analyzer (BioTek, Winooski, VT, USA).

### 2.3 Pathological Specimen Collection and Morphologic Analysis

The dogs were sacrificed at week 28. The hearts were perfused with phosphate-buffered saline (PBS, Hyclone, Logan, UT, USA) and then isolated to measure their weight. To collect the pathological specimen, myocardial tissue blocks of 5 mm × 3 mm × 2 mm were separately gained from left ventricular (LV) and right ventricular (RV) walls. After being fixed with 10% paraformaldehyde (Servicebio Biotech, Wuhan, China) for 24 hours, they were embedded in paraffin (Servicebio Biotech, Wuhan, China), and sliced horizontally into 5 µm slices. Hematoxylin–eosin (HE) staining and Masson’s trichrome staining were performed to analyze the morphological abnormalities and fibrosis, according to the manufacturer’s recommended protocol (Servicebio Biotech, Wuhan, China). Five high-power random fields were chosen from five sections of each heart and quantified in a blinded manner for measurements. The cross-sectional area (CSA) of the cardiomyocytes and the area of the cardiac fibrosis were evaluated separately through morphometric analysis of the HE and Masson’s trichrome staining sections. Specimens were observed under a light microscope (Nikon, Tokyo, Japan) and representative images were selected. The images were measured through an automated image analysis system (Image-Pro Plus 5.0, Bethesda, MD, USA).

### 2.4 Functionally Enrichment and Matrix Correlation

Functionally annotated enrichment analysis was conducted using the Metascape (https://metascape.org/gp/index.html) database [12]. Sangerbox (http://sangerbox.com/about.html) platform was used to analyze and create transcript chord plots and matrix correlation heat maps [13]. Spatial transcriptome and single nucleus RNA sequencing (snRNA SEQ) data were obtained via the STOmicsDB (https://db.cngb.org/stomics/) assets [14].

### 2.5 Real-Time Fluorescence Quantitative Polymerase Chain Reaction (RTFQ-PCR)

To extract mRNA from cells or heart tissue, we used RNAiso Plus#9109 (Takara Bio, Kusatsu, Japan). Next, RNA was reverse transcribed into cDNA using the PrimeScript™ reverse transcription kit# RR036A (Takara Bio, Kusatsu, Japan). The RTFQ-PCR kit (Applied Biosystems, Foster City, CA, USA) was used for the destination analysis. RTFQ-PCR was performed by an ABI 7300 (Thermo Fisher Scientific, Waltham, MA, USA) detection system using the SYBR Green PCR detection method. We prepared 20 µL RTFQ-PCR reaction mixture containing 10 µL qPCR SYBR Green Master Mix (Takara Bio, Kusatsu, Japan), 0.4 µL F primer, 0.4 µL R primer, 1 µL cDNA template, and 1 µL sterile deionized water. The reaction cycle conditions were 95 °C for 30 seconds followed by 40 cycles of 95 °C for 10 seconds and 60 °C for 30 seconds.

### 2.6 Two-Dimensional Echocardiography

Two-dimensional echocardiography was performed using an iE33 echocardiographic system (Philips Medical Systems, Andover, MA, USA) with an S5-1 transducer (1–5 MHz) at baseline (week 0) in both groups, then again after the second infusion (week 4), the fourth infusion (week 8), the sixth infusion (week 12), the eighth infusion (week 16), and three months after the eighth infusion (week 28) (Fig. 1). Two-dimensional echocardiograms were acquired from the parasternal and apical views to monitor regional myocardial function. The transverse diameter of the right ventricular basal segment (RVD1), middle segment (RVD2), and apical segment (RVD3) were collected for analysis. Tricuspid annular plane systolic excursion (TAPSE) was measured by M-mode tracings from the apical four-chamber view. Moreover, mitral inflow was recorded by pulsed-wave Doppler to measure peak velocities of early (E) and late (A) filling; mitral annular early (E’) and late (A’) peak velocities were determined from tissue Doppler imaging.

### 2.7 Real-Time Three-Dimensional Echocardiography 

After examining the 2DE, RT3DE was performed in both groups using the iE33 echocardiographic system equipped with an X3-1 transducer (1–3 MHz). Standard apical four-chamber views were recorded and 3D full-volume dynamic images of three consecutive cardiac cycles were stored for offline analysis by TomTec 4D LV (4.6.0.411, TomTec Imaging Systems GMBH, Unterschleißheim, Germany). The software semiautomatically traced the endocardial contours of the LV and RV at end-diastole and end-systole and allowed for manual modification when needed. The right ventricular end-diastolic volume (RVEDV), right ventricular end-systolic volume (RVESV), right ventricular ejection fraction (RVEF), right ventricular longitudinal free-wall strain (RVLFS), right ventricular longitudinal septal strain (RVLSS), left ventricular end-diastolic volume (LVEDV), left ventricular end-systolic volume (LVESV), left ventricular ejection fraction (LVEF), left ventricular global longitudinal strain (LVGLS) and left ventricular global circumferential strain (LVGCS) were calculated.

### 2.8 Statistical Analysis

Data are expressed as mean ± standard deviation (SD).

The percentage variation was calculated using the following formula:

$$\text{ percentage variation }=\frac{\text{ pre-chemptherapy }\,–\,\text{ post-chemotherapy }}{\text{ pre-chemotherapy }}\times 100\%$$

Statistical significance was determined using SPSS (version 25.0, IBM Corp., Chicago, IL, USA) by paired Student’s *t*-test or one-way ANOVA with Tukey’s post hoc test after applying Benjamini–Hochberg correction for numerical variables. One-way repeated measures ANOVA with Tukey’s post hoc test was used in analyzing related groups data. *p* values and correlation coefficient were calculated using Spearman correlation analysis. GraphPad Prism (version 7.02 for Windows, GraphPad Software, Inc., La Jolla, CA, USA) was utilized to generate the statistical graph. A *p* value < 0.05 was considered statistically significant.

## 3. Results

### 3.1 General Characteristics of the Novel Beagle Dog Model

**Fig. 2.**
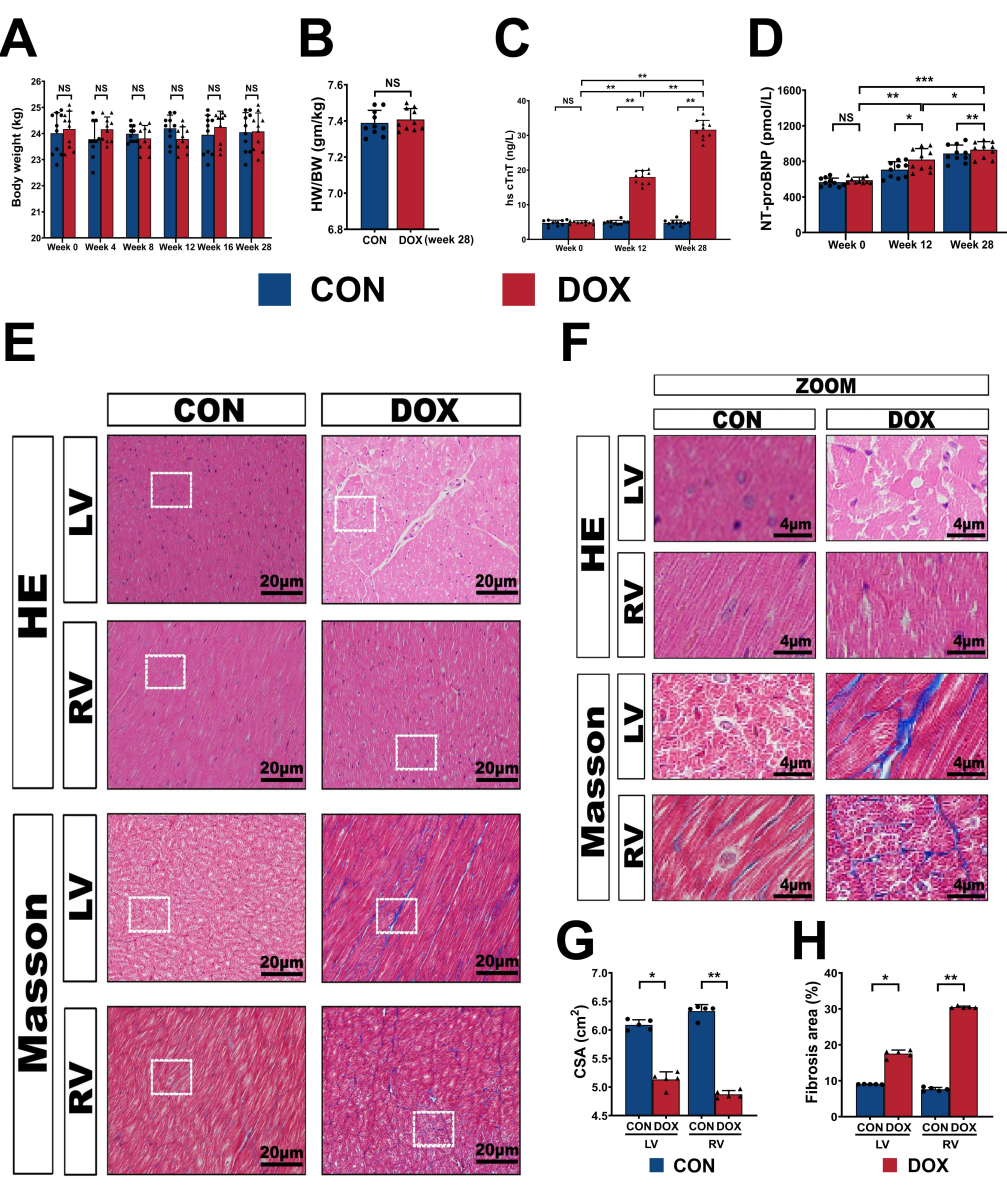
**General characteristics of the novel beagle dog model**. (A,B) Body weight and heart weight/body weight ratio before and after DOX infusion. (C,D) Serum hs cTnT and NT-proBNP levels before and after DOX infusion. (E) Representative HE and Masson’s trichrome staining light micrographs (400 amplification) from CON and DOX groups. (F) Magnified regions from (E). (G,H) Quantification of the cross-sectional area and the area percentages of fibrosis. CON, control group; DOX, doxorubicin group; HW/BW, heart weight/body weight; hs cTnT, high-sensitive serum cardiac troponin T; NT-proBNP, N-terminal pro-brain natriuretic peptide; LV, left ventricle; RV, right ventricle; HE, Hematoxylin–eosin staining; Masson, Masson’s trichrome staining; CSA, cross-sectional area; ZOOM, magnified regions. ⚫ represents data from CON group; ▲ represents data from DOX group. Values are expressed as the mean ± SD. N = 10 dogs per group. *p* values are calculated using paired Student’s t-test or one-way repeated measures ANOVA with Tukey’s post hoc test. NS, no significance; *p < 0.05; **p < 0.01; ***p < 0.001.

The CON group experienced no adverse effects during the whole process. Conversely, three out of ten beagle dogs in the DOX group experienced three to four days of appetite loss and hematochezia after the fourth injection, although they recovered after receiving a soft and low-residue diet and this situation did not affect the fifth injection. To more effectively avoid gastrointestinal hemorrhage, all animals were fasted for 1 day before the following injections and all of them were provided soft and low-residue diets following the injections. All the beagle dogs survived to the end of the experiment without any significant change in weight (vs. week 0 or vs. the CON group at each follow-up, all *p* > 0.05) (Fig. 2A). Heart weight/body weight (HW/BW) showed no obvious differences between the CON and DOX groups at week 28 (*p* > 0.05) (Fig. 2B), while no obvious necrosis was observed at the injection site in the DOX group during the experiment.

To assess the effect of DOX-induced myocardial injury and heart failure, we examined the expression of hs cTnT and NT-proBNP in the serum at weeks 0, 12, and 28. Compared with week 0 (4.92 ± 0.49 ng/L), the serum hs cTnT levels were appreciably elevated at week 12 (17.99 ± 1.92 ng/L) and week 28 (31.63 ± 2.72 ng/L) (*p* < 0.01, respectively). Analogously, it increased significantly at weeks 12 and 28 compared with those in the control group (4.28 ± 0.68 ng/L and 4.78 ± 0.82 ng/L, respectively) at each follow-up (both *p* < 0.01) (Fig. 2C). Moreover, the hs cTnT level at week 28 was higher than at week 12 (*p* < 0.01) owing to the DOX cumulative dose effect, which indicated progressive myocardial damage following the DOX injection. The NT-proBNP showed an upward trend, similar to hs cTnT (all *p* < 0.05) (Fig. 2D).

Necropsy examinations (Fig. 2E) and regional magnification images (Fig. 2F) in the DOX group showed moderate myocardial disease. The degenerative changes observed in the HE staining were characterized by the early to medium multifocal invasion of the myocardium, generation with evaporation of myocardial fibers, areas of fiber disruptors, and scattered inflammatory cell infiltration (Fig. 2F). Similarly, CSA of RV and LV were strongly decreased in the DOX group compared to the CON group (Fig. 2G). Furthermore, DOX caused a marked increase in collagen deposits under Masson’s trichrome staining, which were observed predominantly via the fibrosis of the interstitium in the cardiomyocytes (Fig. 2E,F). In the DOX group, the percentage areas of fibrosis in both the RV and LV increased significantly compared to the control group (Fig. 2H), thereby implying that the myocardium was in the medium stage of the DOX-induced pathological changes.

### 3.2 Doxorubicin Toxicity is Critical for Inflammatory Reprogramming in Myocardium

Due to the absence of spatio-temporalomic data concerning DOX-induced injuries in beagles, we obtained spatial transcriptome data linked to the protective and restorative effects of *Mus musculus* hearts in response to injury via the STOmicsDB (https://db.cngb.org/stomics/) assets, from which we extracted significant single nucleus RNA sequencing (snRNA SEQ) information for further analysis (https://db.cngb.org/stomics/datasets/STDS0000110).

**Fig. 3.**
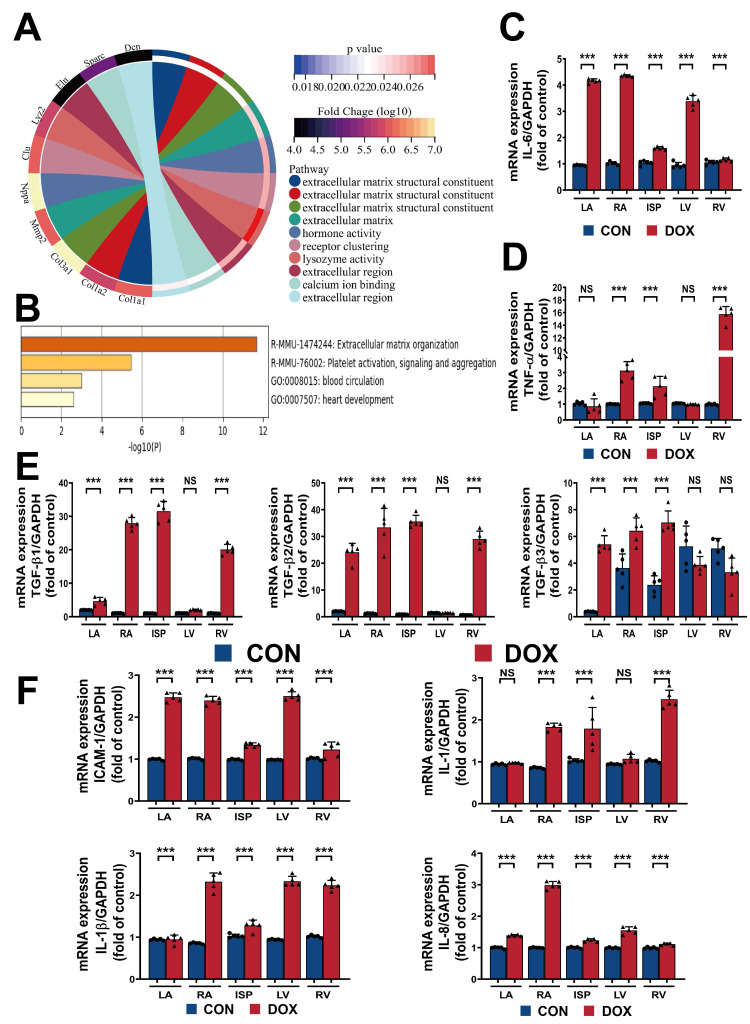
**Doxorubicin toxicity triggers inflammatory reprogramming in the myocardium**. (A,B) The transcript chord plot and the pathway enrichment adapted from STOmicsDB assets and Metascape database. (C,D) Quantification of IL-6 and TNF-α transcript levels normalized to Glyceraldehyde-3-phosphate dehydrogenase (GAPDH) in dog heart sections. (E) Quantification of TGF-β1, TGF-β2, and TGF-β3 transcript levels normalized to GAPDH in dog heart sections. (F) Quantification of ICAM-1, IL-1, IL-1β, and IL-8 transcript levels normalized to GAPDH in dog heart sections. Col1a1, collagen type I alpha 1; Col1a2, collagen type I alpha 2; Col3a1, collagen type III alpha 1; Mmp2, matrix metallopeptidase 2; Nppa, natriuretic peptide type A; Clu, clusterin; Lyz2, lysozyme 2; Eln, elastin; Sparc, secreted acidic cysteine rich glycoprotein; Dcn, decorin; LV, left ventricle; RV, right ventricle; ISP, interventricular septum; LA, left atrium; RA, right atrium; CON, control group; DOX, doxorubicin group; IL-6, interleukin 6; GAPDH, Glyceraldehyde-3-phosphate dehydrogenase; TNF-α, tumor necrosis factor α; TGF-β1, transforming growth factor β1; TGF-β2, transforming growth factor β2; TGF-β3, transforming growth factor β3; ICAM-1, intercellular adhesion molecule 1; IL-1, interleukin 1; IL-1β, interleukin 1β; IL-8, interleukin 8. Values are expressed as the mean ± SD. ⚫ represents data from CON group; ▲ represents data from DOX group. *p* values are calculated using paired Student’s *t*-test or one-way repeated measures ANOVA with Tukey’s post hoc test. NS, no significance; ***p < 0.001.

The transcript chord plot and the enrichment of the related gene pathways (Fig. 3A,B) suggested that changes are concentrated primarily in multiple enzyme-linked receptor protein signaling pathways and collagen metabolism during the process of coping with injury. This observation led us to hypothesize that the interaction between doxorubicin toxicity and myocardial stress could regulate myocardial inflammatory programming and induce various signal pathway imbalances, leading to subsequent collagen metabolism disorders. To assess this hypothesis, we used RTFQ-PCR to measure the transcript levels of hallmark inflammatory genes in the DOX group, including interleukin 6 (IL6) and tumor necrosis factor-alpha (TNFα). The results displayed a noticeable increase in IL6 and TNFα transcript levels in the left and right ventricles and interventricular septum compared to the CON group (Fig. 3C,D). Additionally, we measured the gene expression levels related to intercellular adhesion molecules, leukocyte chemotaxis, and extracellular matrix deposition, including transforming growth factor β (TGFβ), intercellular adhesion molecule 1 (ICAM1), interleukin 1 (IL1), interleukin 1β (IL1β), interleukin 8 (IL8), etc. The measurements demonstrated that these transcripts were also increased in almost all parts of the heart in the DOX group (Fig. 3E,F).

### 3.3 Inflammatory Reprogramming Activation Promotes Myocardial Remodeling

**Fig. 4.**
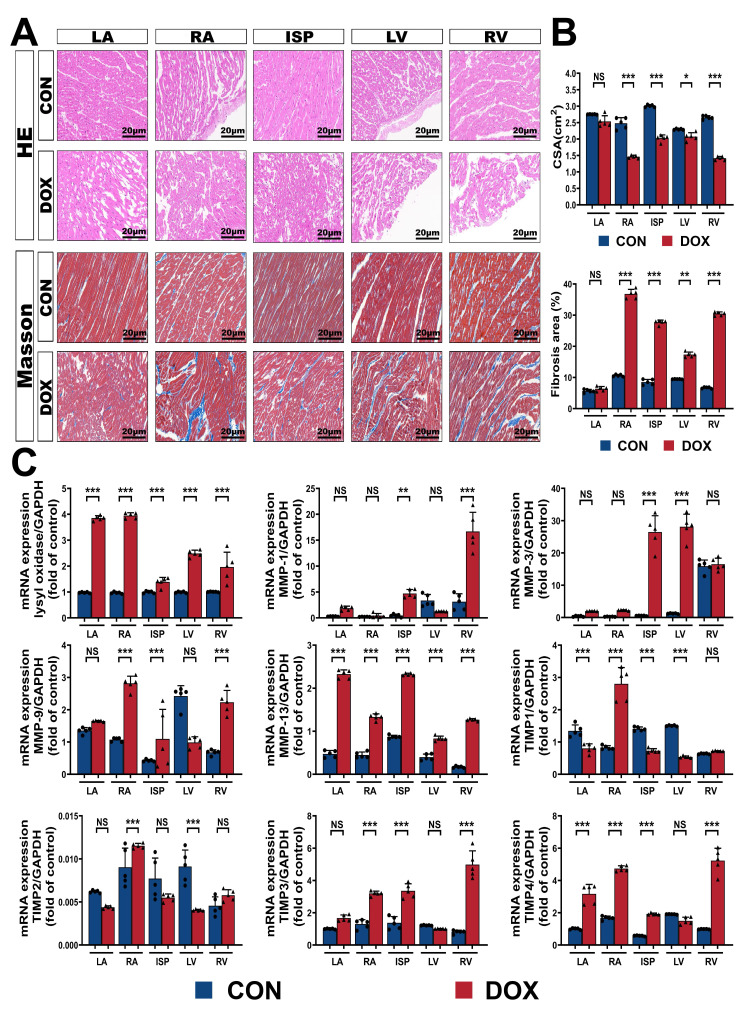
**Inflammatory reprogramming activation contributes to myocardial remodeling in DOX-related cardiomyopathy**. (A) Representative HE and Masson’s trichrome staining light micrographs (400 amplification) from CON and DOX groups. (B) Quantification of the CSA and the percentage areas of fibrosis from (A). (C) Quantification of lysyl oxidase, MMP family, and TIMP family transcript levels normalized to GAPDH in dog heart sections. HE, Hematoxylin–eosin staining; Masson, Masson’s trichrome staining; CON, control group; DOX, doxorubicin group; LV, left ventricle; RV, right ventricle; ISP, interventricular septum; LA, left atrium; RA, right atrium; CSA, cross-sectional area; GAPDH, Glyceraldehyde-3-phosphate dehydrogenase; MMP-1, matrix metalloproteinase 1; MMP-3, matrix metalloproteinase 3; MMP-9, matrix metalloproteinase 9; MMP-13, matrix metalloproteinase 13; TIMP-1, tissue inhibitor of matrix metalloproteinase 1; TIMP-2, tissue inhibitor of matrix metalloproteinase 2; TIMP-3, tissue inhibitor of matrix metalloproteinase 3; TIMP-4, tissue inhibitor of matrix metalloproteinase 4. Values are expressed as the mean ± SD. ⚫ represents data from CON group; ▲ represents data from DOX group. *p* values are calculated using paired Student’s *t*-test or one-way ANOVA. NS, no significance; *p < 0.05; **p < 0.01; ***p < 0.001.

In the DOX group, HE and Masson’s trichrome staining revealed cardiomyocyte structural disorder, patchy interstitial inflammatory infiltration, and obvious interstitial collagen deposition in various parts of the myocardium (Fig. 4A). The statistical results confirmed the occurrence of myocardial structural remodeling under the effect of informatory reprogramming. Specifically, the CSA in the DOX group cardiomyocytes decreased significantly at all points in the heart, and the percentage area of fibrosis increased significantly (all *p* < 0.05) (Fig. 4B). Key transcripts of collagen metabolism and deposition, including lysyl oxidase, matrix metalloproteinase (MMP) family, and tissue inhibitor of matrix metalloproteinase (TIMP) family were analyzed. The results revealed a significant upward trend in these transcripts after DOX administration at most points in the heart (Fig. 4C), which was consistent with the characteristics of the pathological examination.

### 3.4 Myocardial Remodeling and Volume Changes Influence the Three-Dimensional Strain Capacity and Predominantly Affect the Right Ventricle

**Fig. 5.**
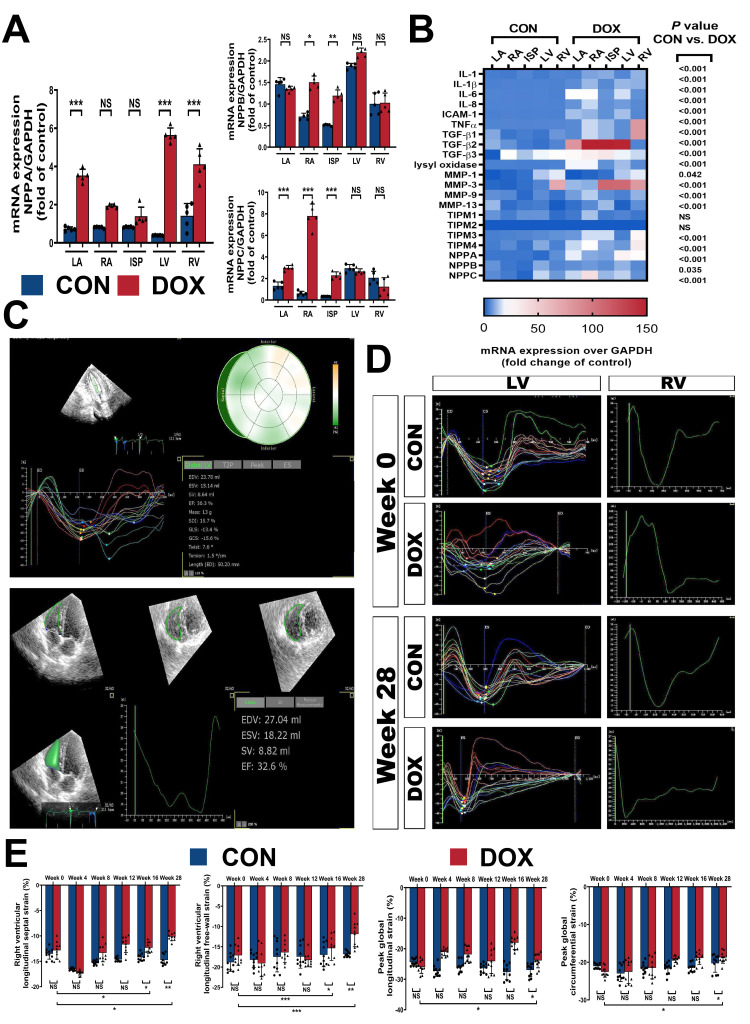
**Myocardial remodeling and volume changes influence the 3D strain capacity**. (A) Quantification of NPPA, NPPB, and NPPC transcript levels normalized to GAPDH in dog heart sections. (B) Heat map of inflammation, collagen metabolism, and volume load in the right- or left side of the heart in the CON and DOX groups. (C) Examples of 3D echocardiographic studies for the LV (above) and RV (below) strain parameters using TomTec offline analysis software. (D) Three-dimensional echocardiographic studies of LV and RV strain curves at baseline (week 0) and endpoint (week 28) using TomTec offline analysis software. (E) Quantification of 3D echocardiographic parameters for RVLSS, RVLFS, LVGLS, and LVGCS. LV, left ventricle; RV, right ventricle; ISP, interventricular septum; LA, left atrium; RA, right atrium; NPPA, natriuretic peptide A; NPPB, natriuretic peptide B; NPPC, natriuretic peptide; GAPDH, Glyceraldehyde-3-phosphate dehydrogenase; CON, control group; DOX, doxorubicin group; IL1, interleukin 1; IL1β, interleukin 1β; IL6 interleukin 6; IL8 interleukin 8; ICAM1, intercellular adhesion molecule 1; TNFα, tumor necrosis factor α; TGFβ1, transforming growth factor β1; TGFβ2, transforming growth factor β2; TGFβ3, transforming growth factor β3; MMP1, matrix metalloproteinase 1; MMP3, matrix metalloproteinase 3; MMP9, matrix metalloproteinase 9; MMP13, matrix metalloproteinase 13; TIMP1, tissue inhibitor of matrix metalloproteinase 1; TIMP2, tissue inhibitor of matrix metalloproteinase 2; TIMP3, tissue inhibitor of matrix metalloproteinase 3; TIMP4, tissue inhibitor of matrix metalloproteinase 4; RVLSS, right ventricular longitudinal septal strain; RVLFS, right ventricular longitudinal free-wall strain; LVGLS, left ventricular global longitudinal strain; LVGCS, left ventricular global circumferential strain. Values are expressed as the mean ± SD. ⚫ represents data from CON group; ▲ represents data from DOX group. *p* values are calculated using paired Student’s *t*-test or one-way ANOVA. NS, no significance; *p < 0.05; **p < 0.01; ***p < 0.001.

Inflammatory cascades, fibrosis, and myocardial remodeling frequently cause changes in ventricular and circulatory capacity, leading to a close association with the natriuretic peptides family (NPS). This family plays a vital role in natriuretic, diuretic, vasodilator, anti-sympathetic, and renin–aldosterone inhibiting activities. Under this premise, we detected the natriuretic peptide A (NPPA) transcription levels in this family, as well as natriuretic peptide B (NPPB) and natriuretic peptide (NPPC), which play similar roles as key markers of ventricular volume load change in response to inflammation and fibrosis. Unlike inflammatory reprogramming, which was widely upregulated in almost all parts of the heart, the NPPA, NPPB, and NPPC transcript levels tended to change significantly for the right side and interventricular septum (Fig. 5A), suggesting that further effects of myocardial remodeling varied in different parts of the heart. To judge the impact of DOX on typical parts in terms of inflammation, collagen metabolism, and volume load more intuitively and comprehensively, we analyzed their multidimensional relationships by thermogram. It could be seen that DOX-related myocardial toxicity and changes appeared to play a more important role in the right ventricle, followed by the interventricular septum, whereas there were only a few significant changes in the left heart (Fig. 5B).

The three-dimensional speckle tracking method could more accurately and dynamically reconstruct the cardiac chamber structure and has irreplaceable advantages in evaluating the strain capacity and cardiac function of large animals. Therefore, we used three-dimensional echocardiography to record the typical endocardial map of the left and right ventriculares during the cardiac cycle and combined it with the corresponding interpolation function to calculate the fitting curve and key parameters (Fig. 5C). Fig. 5D illustrates the standard left and right ventricular strain curves in the control and DOX groups at baseline (week 0) and endpoint (week 28).

Significant strain abnormalities were observed in the RVLSS, RVLFS, LVGLS, and LVGCS at week 28 (vs. week 0 or CON group, *p* < 0.05, respectively), which are suggestive of a DOX-related late ventricular strain impairment. To be more specific, for RVLSS and RVLFS, the decline was also significant at week 16 compared with week 0 (–10.98 ± 2.16% vs. –13.04 ± 4.09% and –15.15 ± 1.71 vs. –17.46 ± 1.73, both *p* < 0.05) (Fig. 5E), which appeared earlier than in the left ventricular areas.

Hence, a correlation between myocardial remodeling, volume load adjustments, and alterations in ventricular strain capacity could be detected. Over the observation period, the cardiac damage caused by DOX led to modifications in the three-dimensional cardiac strain capacity of beagles, notably in the right ventricle.

### 3.5 Mutual Promotion of Myocardial Remodeling and Strain Disorders Can Induce Right Ventricular Functional Imbalance

**Fig. 6.**
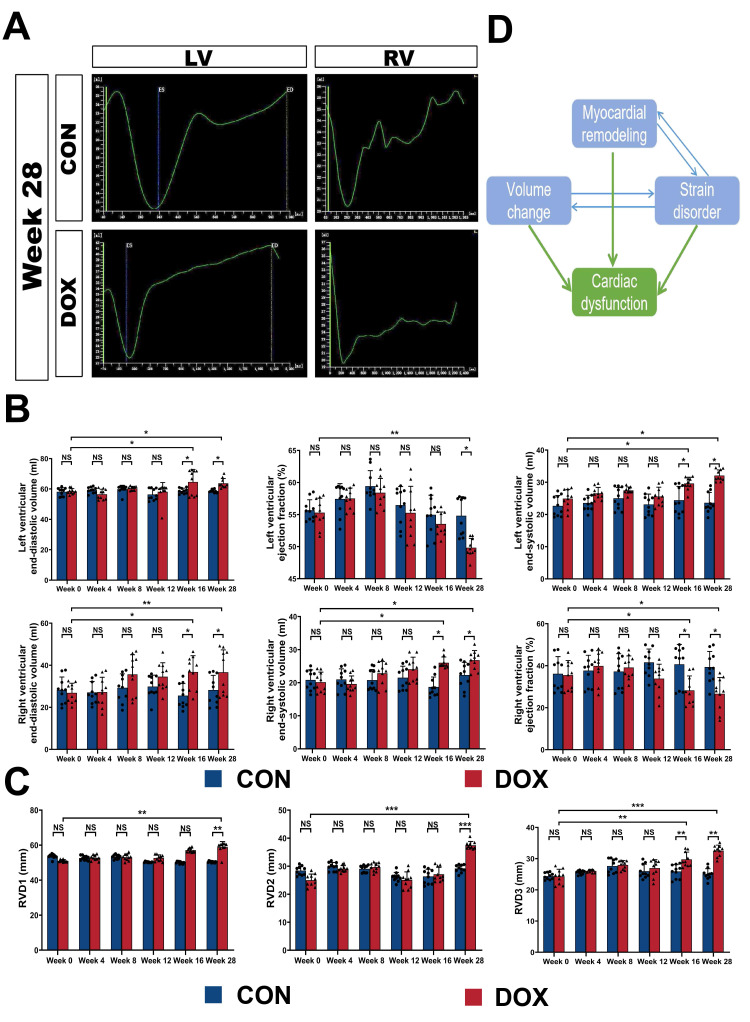
**Myocardial remodeling and strain disorders influence the 3D cardiac function**. (A) Examples of 3D echocardiographic studies of LV and RV function curves using TomTec offline analysis software. (B) Quantification of 3D echocardiographic parameters for LVEDV, LVESV, LVEF, RVEDV, RVESV, and RVEF in the CON and DOX groups. (C) Quantification of 3D echocardiographic parameters for transition diameters in the right ventricular basal (RVD1), middle (RVD2), and apical (RVD3) segments in the CON and DOX groups. (D) The relationship among myocardial remodeling, volume load, strain disorder, and cardiac dysfunction. LV, left ventricle; RV, right ventricle; CON, control group; DOX, doxorubicin group; LVEDV, left ventricular end-diastolic volume; LVESV, left ventricular end-systolic volume; LVEF, left ventricular ejection fraction; RVEDV, right ventricular end-diastolic volume; RVESV, right ventricular end-systolic volume; RVEF, right ventricular ejection fraction; RVD1, transition diameters of the right ventricular basal segment; RVD2, transition diameters of the right ventricular middle segment; RVD3, transition diameters of the right ventricular apical segment. N = 10 dogs per group. Values are expressed as the mean ± SD. ⚫ represents data from CON group; ▲ represents data from DOX group. *p* values were calculated using paired Student’s *t*-test or one-way ANOVA. NS, no significance; *p < 0.05; **p < 0.01; ***p < 0.00.

Previous literature has reported a comprehensive regulatory relationship between myocardial remodeling and strain disorder that can mutually reinforce and exacerbate circulatory challenges [15], both of which significantly impact cardiac function. Given the evidence of remodeling and strain abnormalities, as well as their potential connections in a canine model, we aimed to investigate their effect on cardiac function. Specifically, the investigation aimed to determine whether remodeling and strain abnormalities correlated to changes in cardiac function, which is critical in understanding the clinical features of doxorubicin cardiomyopathy. The important parameters of left and right ventricular systolic function were measured and analyzed from week 0 to week 28 using Tomtec software (Fig. 6A).

Compared to week 0 or for each in the CON group, both of RVEDV and RVESV were considerably increased at week 16 and persisted until week 28 (all *p* < 0.05), while LVEDV and LVESV also rose remarkably at week 16 and 28 (vs. week 0 or control group, *p* < 0.05, respectively) (Fig. 6B). More importantly, a significant decrease was noted for RVEF at week 16 (vs. week 0, 33.92 ± 3.59% vs. 38.58 ± 3.58%, *p* < 0.05), which occurred 12 weeks earlier than the decline in LVEF at week 28 (vs. week 0, 49.02 ± 2.07% vs. 54.26 ± 4.38%, *p* < 0.01). The percentage variation observed at week 28 was 31.26% for RVEF and 9.65% for LVEF (the average reduction was 12.06% and 5.24%, correspondingly) (Fig. 6B), indicating that cumulative doses of DOX led to considerable impaired cardiac contractility, which could happen at an earlier stage and develop to a more advanced extent in RV.

The transition diameters of the right ventricular basal (RVD1), middle (RVD2), and apical (RVD3) segments were also reflective of the lateral systolic function by the right heart, thereby exhibiting a similar trend. The results demonstrated that RVD3 increased significantly from week 16 (vs. control group, *p* < 0.01), while the upward trend of converse diameter had affected all right ventricular segments by week 28 (vs. control group, *p* < 0.01) (Fig. 6C).

In the present study, the LV diastolic function and tricuspid annular plane systolic excursion (TAPSE) were explored via 2D Doppler echocardiography. In Table 1, no significant change was found in E, A, E’, and A’ (*p* > 0.05, respectively). Further, we calculated the ratio of E/A and E/E’ and again observed no statistical difference. Similarly, our study detected no statistically significant differences in TAPSE, which was used as an indicator of RV longitudinal systolic function.

**Table 1.** **Parameters of left ventricular diastolic function and right ventricular function at different time points**.

Variables	Week 0		Week 4		Week 8		Week 12		Week 16		Week 28	
	CON	DOX	CON	DOX	CON	DOX	CON	DOX	CON	DOX	CON	DOX
E (cm/s)	51.87 ± 6.21	52.69 ± 7.22	52.79 ± 8.26	53.46 ± 9.66	49.05 ± 2.89	48.96 ± 3.65	53.90 ± 8.32	55.14 ± 9.97	58.92 ± 8.29	58.70 ± 10.09	56.42 ± 9.29	55.30 ± 11.98
A (cm/s)	54.20 ± 5.29	53.08 ± 11.45	51.01 ± 9.87	50.35 ± 12.21	46.83 ± 7.29	47.06 ± 8.12	49.91 ± 8.20	50.75 ± 11.60	52.19 ± 8.14	50.64 ± 9.56	55.92 ± 9.27	55.50 ± 10.65
E’ (cm/s)	11.70 ± 4.31	12.57 ± 4.45	11.27 ± 0.89	10.74 ± 1.68	11.15 ± 2.02	10.63 ± 2.93	10.32 ± 2.02	9.95 ± 1.84	11.08 ± 2.19	10.76 ± 3.69	11.86 ± 1.76	11.11 ± 2.49
A’ (cm/s)	16.43 ± 3.27	16.04 ± 3.69	14.83 ± 4.92	15.40 ± 5.58	15.46 ± 2.10	14.48 ± 3.67	13.63 ± 2.64	14.71 ± 4.04	13.12 ± 4.58	13.71 ± 6.33	16.93 ± 4.27	17.36 ± 6.99
E/A ratio	1.47 ± 0.13	1.05 ± 0.27	1.29 ± 0.47	1.13 ± 0.32	1.15 ± 0.19	1.13 ± 0.25	1.21 ± 0.23	1.14 ± 0.25	1.19 ± 0.24	1.24 ± 0.29	1.10 ± 0.23	1.04 ± 0.25
E/E’ ratio	5.18 ± 2.01	4.72 ± 1.88	4.96 ± 0.84	5.09 ± 1.12	5.03 ± 1.17	4.87 ± 1.08	5.37 ± 1.72	5.73 ± 1.59	5.91 ± 1.72	6.06 ± 2.28	5.86 ± 1.27	5.16 ± 1.46
TAPSE (mm)	19.80 ± 1.00	20.90 ± 1.37	22.10 ± 1.82	21.40 ± 2.07	22.30 ± 1.26	21.70 ± 1.64	20.70 ± 0.93	20.50 ± 1.08	19.60 ± 1.52	19.80 ± 1.48	21.70 ± 0.86	21.10 ± 1.73

Data are expressed as the mean ± SD. E, peak velocities of early transmitral flow; A, peak velocities of late transmitral flow; E’, mitral annular early peak velocity; A’, mitral annular late peak velocity; TAPSE, tricuspid annular plane systolic excursion. N = 10 dogs per group. CON, control; DOX, doxorubicin.

Collectively, these findings suggest that during the observation period, there is an alteration in cardiac function indicators in response to doxorubicin. The transversal systolic function had been affected initially, primarily on the right ventricle. Effects on diastolic and longitudinal systolic functions were relatively small or might occur later.

Considering the comprehensive relationship between myocardial remodeling, strain, and cardiac function, we proposed the following hypothesis: In adriamycin cardiomyopathy, the remodeling of the myocardium itself and the change in the volume load of the chamber jointly led to the change in myocardial strain capacity, while the strain disorder further aggravated the process of myocardial remodeling and the increase in the volume load. The interaction of the two finally contributed to the imbalance in cardiac function (Fig. 6D).

### 3.6 Statistical Correlation between DOX-induced Inflammatory Reprogramming and the Changes in Strain Capacity and Cardiac Function

**Fig. 7.**
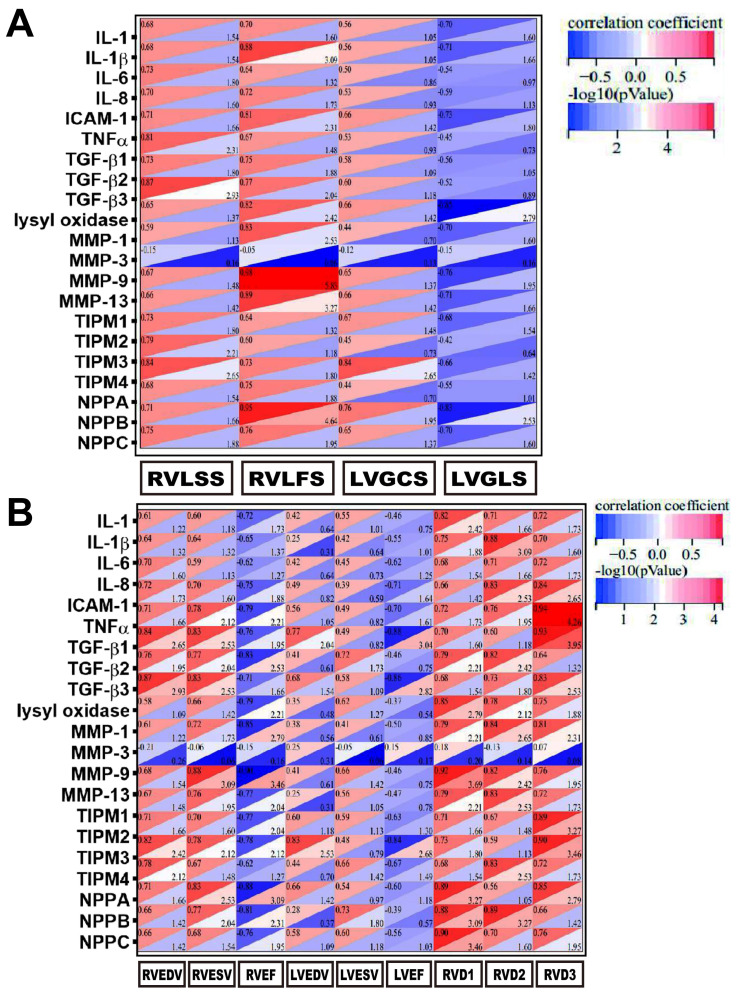
**Statistical correlation between inflammatory reprogramming, strain capacity, and cardiac function**. (A) Correlation matrix thermograms between inflammatory reprogramming and strain disorders. (B) Correlation matrix thermograms between inflammatory reprogramming and cardiac dysfunction. IL1, interleukin 1; IL1β, interleukin 1β; IL6, interleukin 6; IL8 interleukin 8; ICAM1, intercellular adhesion molecule 1; TNFα, tumor necrosis factor α; TGFβ1, transforming growth factor β1; TGFβ2, transforming growth factor β2; TGFβ3, transforming growth factor β3; MMP1, matrix metalloproteinase 1; MMP3, matrix metalloproteinase 3; MMP9, matrix metalloproteinase 9; MMP13, matrix metalloproteinase 13; TIMP1, tissue inhibitor of matrix metalloproteinase 1; TIMP2, tissue inhibitor of matrix metalloproteinase 2; TIMP3, tissue inhibitor of matrix metalloproteinase 3; TIMP4, tissue inhibitor of matrix metalloproteinase 4; NPPA, natriuretic peptide A; NPPB, natriuretic peptide B; NPPC, natriuretic peptide; RVLSS, right ventricular longitudinal septal strain; RVLFS, right ventricular longitudinal free-wall strain; LVGCS, left ventricular global circumferential strain; LVGLS, left ventricular global longitudinal strain; RVEDV, right ventricular end-diastolic volume; RVESV, right ventricular end-systolic volume; RVEF, right ventricular ejection fraction; LVEDV, left ventricular end-diastolic volume; LVESV, left ventricular end-systolic volume; LVEF, left ventricular ejection fraction; RVD1, transition diameters of the right ventricular basal segment; RVD2, transition diameters of the right ventricular middle segment; RVD3, transition diameters of the right ventricular apical segment. *p* values and correlation coefficient are calculated using Spearman correlation analysis.

Although the above results provided compelling evidence that the effects on cardiac strain and function by DOX relied on subsequent cascade reactions in inflammatory reprogramming, the correlation between them lacked statistical support, meaning it needs to be further explored in a multidimensional context. Thus, we investigated the relationship between reprogramming-related transcript changes, 3D strain capacity, and cardiac function, which were visualized by correlation matrix thermograms (Figs. 7A,6B). The gram between inflammatory reprogramming and strain capacity revealed that, firstly, the right ventricular strain damages strongly correlated with inflammatory reprogramming (most Spearman’s coefficients ≥0.70). Secondly, there was a weak correlation (Spearman’s coefficients from 0.45 to 0.65) between left ventricular circumferential strain injury and reprogramming, while left ventricular longitudinal strain appeared to resist inflammation-induced injuries (Fig. 7A), which has rarely been reported before. The correlation matrix between reprogramming and cardiac function demonstrated that inflammation aggravation strongly positively correlated with left and right ventricular end-diastolic and systolic volumes, while it was negatively associated with LVEF and RVEF. Interestingly, Spearman’s coefficients between inflammation and right ventricular functional indexes (0.60 to 0.90) were generally greater than those for the left ventricular (0.30 to 0.65), indicating a more pronounced relationship between right ventricular function and inflammation. Moreover, RVD1, RVD2, and RVD3 were mainly positively correlated with inflammatory reprogramming, with a correlation coefficient ranging from 0.70 to 0.90 (Fig. 7B). In summary, our data reaffirms that inflammatory reprogramming was a key regulator of right ventricular transverse systolic function.

### 3.7 Inflammatory Reprogramming Mediates the Changes in Three-Dimensional Strain Capacity and Cardiac Function in DOX-Induced Cardiomyopathy Beagle Dog Model 

**Fig. 8.**
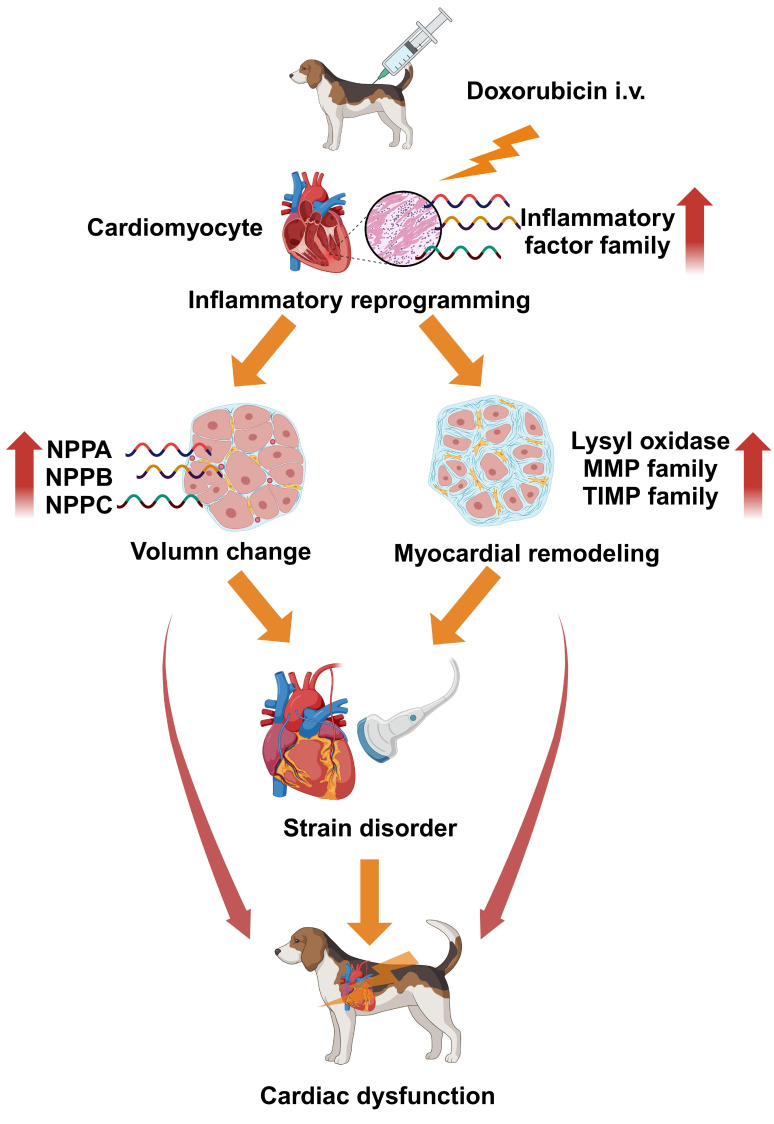
**Inflammatory reprogramming mediated disorders in strain capacity and cardiac function in the DOX-related cardiomyopathy beagle dog model**. (created with https://www.biorender.com/). i.v., intravenous administration; NPPA, natriuretic peptide A; NPPB, natriuretic peptide B; NPPC, natriuretic peptide; MMP, matrix metalloproteinase; TIMP, tissue inhibitor of matrix metalloproteinase; DOX, doxorubicin.

The disease process in the novel DOX-induced cardiomyopathy beagle dog model has been summarized in Fig. 8. Doxorubicin myocardial toxicity extensively activated and upregulated the inflammatory reprogramming process, causing myocardial remodeling and changes in cardiac volume load. Under dual pressure, myocardial strain capacity became disorderly, resulting in a disrupted state of cardiac function, which finally cascaded into functional imbalance.

## 4. Discussion

DOX still plays a crucial role in tumor treatment, despite it causing long-term and serious side effects, via myocardial injuries [1]. Currently, there is no consensus as to the mechanism related to the onset of myocardial injury owing to the lack of any accurate experimental models [7, 16]. Therefore, cardio-oncology calls for an efficient and stable new model to assist clinical exploration.

Due to the obscurity and refractoriness of congestive heart failure caused by DOX, precise and systematic early monitoring is required, with techniques such as echocardiography, magnetic resonance imaging (MRI), and single-photon emission-computed tomography (SPECT) [5, 6]. However, using small animal models, such as mice, rats, and rabbits, to measure these indicators is limited due to the significant structural deviations between their hearts and humans [2, 17, 18]. Beagle dogs, on the other hand, are considered a more suitable model because their heart characteristics are similar to humans.

However, the currently utilized beagle model, which involves DOX being infused into the coronary artery, requires surgical methods, such as bilateral thoracotomy [19], or the insertion of a femoral artery catheter using a fluoroscope [7]. Both operation techniques require a high level of skill and have mortality rates that exceed 40%. Intravenous injection seems to be a more convenient and practical method. Inspired by the study by Cheng *et al*. [20], our final plan administered only 1.5 mg/kg of DOX every two weeks, at a concentration of 0.4 mg/mL, with an injection duration of 30 to 45 minutes. Additionally, omeprazole and Bacillus were added at a 1:1 ratio as gastrointestinal protective agents to prevent the fatal gastrointestinal bleeding caused by DOX, which was observed in our previous experiments (unpublished data: **Supplementary Fig. 1**).

Our new protocol has also led to a low-mortality DOX-induced cardiotoxicity beagle dog model, characterized by cardiac dysfunction, right and left ventricular strain impairment, and myocardial disorders.

Multiple indicators were used during the model evaluation. An example is hs cTnT, which can predict myocardial injury in clinical practice [21, 22]. Similar to the findings of Cove-Smith *et al*. [23], our study observed an increase in hs cTnT levels that correlated positively with DOX after a cumulative dose of 9 mg/kg and preceded the detection of echocardiographic abnormalities, which were present only in doses higher than 12 mg/kg. Similar trends were observed in NT-proBNP levels, although the magnitudes of changes were comparatively smaller. This finding might be partially due to a delayed response of NT-proBNP to changes in cardiac volume and pressure resulting from the self-compensatory effects seen in the early-to-mid stages of cardiac dysfunction [24].

Our study suggested that DOX triggers inflammatory reprogramming, resulting in a proinflammatory state and myocardial remodeling. By referring to the spatial transcriptome from mice coping with injury-induced stress, the main upregulated genes were involved in collagen metabolism and enzyme-linked receptor protein signaling pathways, we assumed that broad signaling disorders can lead to unbalanced collagen metabolism.

The present experiment demonstrated an extensive upregulation in proinflammatory factors, including IL6 and TNFα, and various inflammatory regulatory factors, indicating that inflammatory reprogramming played a crucial role in this imbalance process. Reprogramming led to a shift towards a proinflammatory state *in vivo*, resulting in multidimensional reactions. Firstly, the upregulation of various MMPs and their tissue inhibitors (TIMPs) resulted in collagen accumulation and myocardial fibrosis, as confirmed by Masson staining of the left and right atria, ventricles, and interventricular septa. Additionally, myocardial remodeling triggers changes in the volume load of each chamber, which consequently affects the expression of the brain natriuretic peptide family genes.

Myocardial remodeling and changes in volume load can result in alterations to the three-dimensional strain capacity. By heat mapping all inflammation, fibrosis, and brain natriuretic peptide transcripts, we found that transcriptional upregulation tended to be more pronounced in the right heart than in the left. Since structural disorders are often accompanied by functional alterations, we posited that misalignments in echocardiographic indicators may arise in respective spatial locations.

RT3DE has been proven to yield plentiful information on cardiac strain parameters as well as provide more accurate evaluations of LVEF and RVEF than 2DE [25]. However, in terms of ventricular strain, it is noteworthy that RVLFS and RVLSS began to decline as early as week 16; earlier than LVGLS and LVGCS, which declined at week 28. The asynchronous changes in strain may be due in part to the thickness of the ventricular wall, which affects wall stress. According to Laplace’s law, wall stress increases with pressure and radius but decreases with wall thickness [26, 27]. Considering that the right ventricular wall is thinner than the left, it experiences greater wall stress and appears more susceptible to spatial strain degradation.

Myocardial remodeling is a factor that affects cardiac function by altering the myocardial fiber properties during systole and diastole [10], whereas strain disorder disrupts the balance of the regulatory effects, which are multi-segmental and multi-temporal [28]. In terms of systolic function, LVEF remained relatively stable over the initial 16-week period but experienced a significant and sustained decline from week 16 to week 28. In contrast, RVEF demonstrated more significant DOX-induced impairment at an earlier time point (week 16), with a higher percentage variation (13.27% for RVEF vs. 5.26% for LVEF at week 28). To further support this finding, the right ventricular systolic function was assessed using the transverse diameters of the right ventricular basal, middle, and apical segments (RVD1, RVD2, and RVD3), all of which were notably higher than the baseline values and indicative of myocardial toxicity at an early stage. Specifically, this interesting finding was novel in the canine model, as prior studies had not commonly observed early anomalies in the right ventricular systolic function. Prior research by Nagata *et al*. [29] proposed that contractile impairment could be compensated for until DOX exposure resulted in decompensated cardiotoxicity and symptomatic heart failure. It is hypothesized that the thinner right ventricular wall may provide fewer compensation reserves and yield earlier manifestations of reduced RVEF. In contrast, the indices for diastolic function did not exhibit significant changes during the follow-up period, implying that DOX-induced myocardial toxicity may have limited effects on these indicators, or they may experience a late-stage injury.

To investigate the correlation among strain capacity, cardiac function, inflammatory reprogramming, and myocardial remodeling, we utilized multiple correlation matrix thermograms to depict the relationship intuitively. Here, extensive inflammation and remodeling had a greater correlation to the right ventricular strain and function parameters. Moreover, we discovered that DOX toxicity had a minimal impact on the left and right ventricular longitudinal contractions or strain capacities, or that they might experience a time-lag effect. Hence, we need to prolong the follow-up to observe any long-term changes.

In summary, our study reviews the critical role of inflammatory reprogramming in promoting changes to the three-dimensional strain capacity and cardiac function in beagle dogs during the development of DOX-related cardiomyopathy.

One limitation of this study was that the potential late-occurring diastolic and TAPSE abnormalities by DOX were not evaluated. A previous study showed that impaired diastolic function occurred in 60% of breast cancer participants treated with DOX by 1 year, 70% by 2 years, and 80% by 3 years [30], thereby demonstrating that diastolic dysfunction develops gradually over time and TAPSE abnormality might also be delayed. For that reason, follow-ups of over 1 year are also needed for the beagle dog model in the future to ensure that myocardial stiffness reaches the degree where it can be detected by echocardiography.

Another limitation to consider is that all myocardial pathological changes in the study are still in the early to medium stages. Considering this, follow-up experiments can use increased dosages and extend the course of DOX administration to discover more evident variations in the phenotype and their relationship with inflammatory reprogramming.

## 5. Conclusions

We have successfully established a stable DOX-induced myocardial toxicity canine model with low mortality, which was characterized by increased hs cTnT and NT-proBNP levels alongside predominantly right ventricular systolic and strain dysfunction. Inflammatory reprogramming is believed to be one of the initial causes during the myocytic injury process. Therefore, our findings provide a valuable foundation for further research on the mechanisms involved. Ultimately, such initiatives are essential for ensuring humane animal treatment in research settings.

## Data Availability

All data points generated or analyzed during this study are included in this article and there are no further underlying data necessary to reproduce the results.

## Supplementary Material

Supplementary material associated with this article can be found, in the online version, at https://doi.org/10.31083/j.rcm2502062.
