# Supplementary material for: Inflammatory Reprogramming Mediates Changes in Three-Dimensional Strain Capacity and Cardiac Function in Beagle Dogs with Doxorubicin-Related Cardiomyopathy
Source: Rev Cardiovasc Med. 2024 Feb 18;25(2):62. doi: 10.31083/j.rcm2502062 (PMC11263182; doi:10.31083/j.rcm2502062)
Supplement: Supplementary file 1 [file 2153-8174-25-2-062-s1.docx]

**Supplementary Fig. 1**


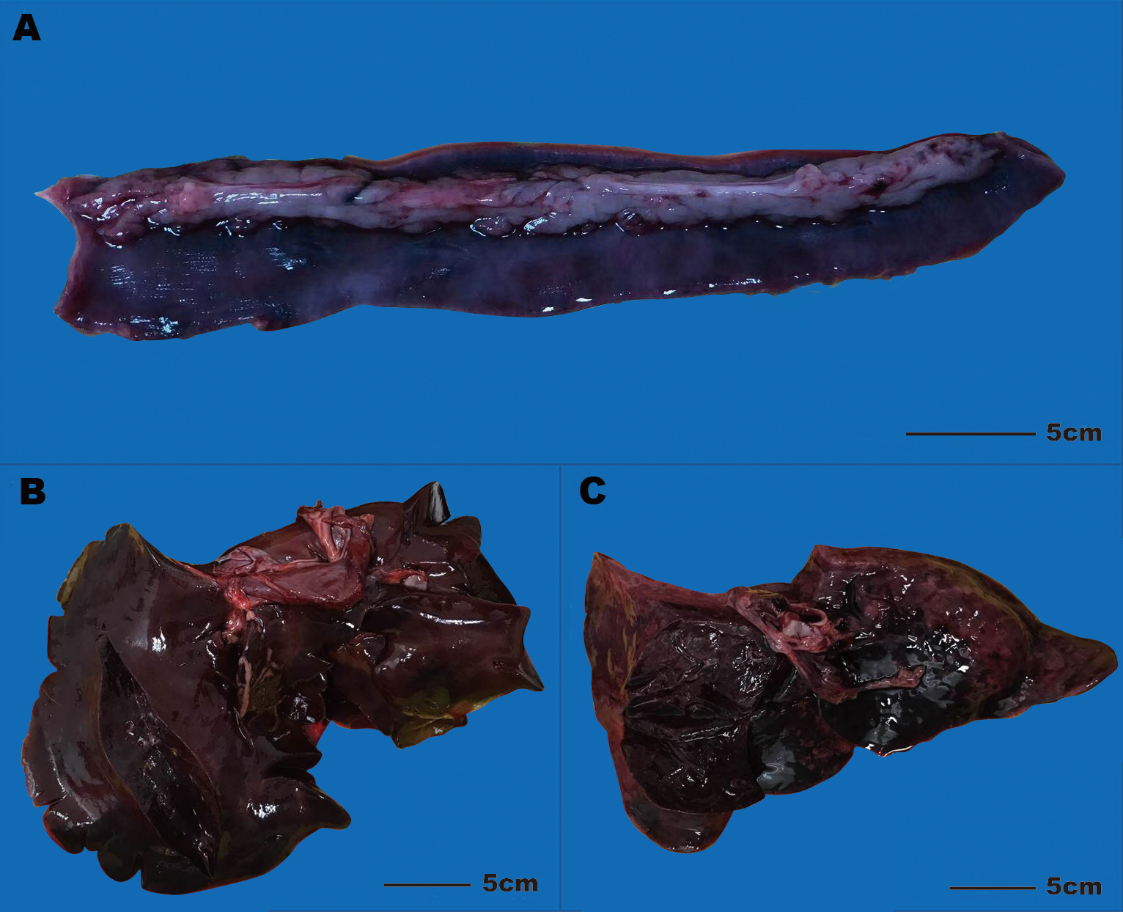


**Supplementary Fig. 1**

Massive hemorrhage points in intestines (A), liver (B) and lungs (C) after the fourth to fifth administration of DOX.
